# Synergistic bactericidal activity of a naturally isolated phage and ampicillin against urinary tract infecting *Escherichia coli* O157 

**DOI:** 10.22038/IJBMS.2019.37561.8989

**Published:** 2020-02

**Authors:** Zahra Moradpour, Nesa Yousefi, Dorna Sadeghi, Abdollah Ghasemian

**Affiliations:** 1 Department of Pharmaceutical Biotechnology, Faculty of Pharmacy, Urmia University of Medical Sciences, Urmia, Iran; 2 Cellular and Molecular Sciences Research Center, Urmia University of Medical Sciences, Urmia, Iran

**Keywords:** Antibiotic resistance, Escherichia coli O157, Lytic phage, Phage therapy, Synergy, Ampicillin

## Abstract

**Objective(s):**

Bacteriophages are infectious replicating entities that are under consideration as antimicrobial bioagents to control bacterial infections. As an alternative or supplement to antibiotics, bacteriophages can be used to circumvent the resistance to existing antibiotics. The aim of this study was to assess the synergistic effect of a naturally isolated phage and ampicillin against *Escherichia coli* O157.

**Materials and Methods:**

In the present study, a natural phage against *E. coli* O157 was isolated, the morphology and molecular characteristics of the phage were identified, and the combination of bacteriophage and antibiotic to combat clinically isolated drug-resistant *E. coli *O157 was evaluated.

**Results:**

The results showed the synergistic action between a naturally isolated phage and ampicillin in solid (disk diffusion test) and liquid culture media. Addition of the isolated phage, gT0*E.co*-MGY2, to the microbial lawn of bacteria in modified antibiotic disk diffusion test, altered susceptibility pattern of *E. coli *O157 from resistant to sensitive based on the inhibition zones. Combinations of bacteriophage and ampicillin significantly enhanced the killing of bacterial strains when compared to treatment with ampicillin or phage alone in liquid culture. Moreover, it lasted few hours for ampicillin to reverse the growth of *E. coli *O157, while the bacteriophage and combination treatment stopped the proliferation of bacteria from the beginning, and this can compensate the delayed onset of antibiotic action.

**Conclusion:**

The synergistic action of bacteriophages and antibiotics is an alternative that cannot only be effective against bacterial infections but also contribute to the reduction of antibiotic resistance.

## Introduction

Bacteriophages (phages), as valuable and well-tolerated alternatives to antibiotics, have been exploited to control foodborne pathogens like *Escherichia* *coli* O157 since their discovery (1-4). Lytic phages land on the host bacteria, multiply inside the microbe to produce the virus progeny and then lyse the cell to re-enter other available cells and repeat the cycle (5). They have frequently been used to treat infectious diseases from the World War II. However, the potential role of them as biotherapeutic agents to combat against resistant bacterial strains has recently been reemphasized by researchers and different global regulatory authorities (6, 7). 

Despite the significant outcome in combating resistant bacteria using phage-mediated biocontrol, phage-resistant strains arise particularly when they are used individually (8). Therefore, phage-antibiotic combination and their interactions with the host has steadily gained tremendous interest (9-12). A better understanding of these interactions seems inevitable if the phages are to be a class of antibacterial therapeutics. Combination therapies may be more beneficial than a single component for preventing or limiting the evolution of resistant strains and can be introduced as a solution for the rapid increase of ineffective clinically essential antibiotics. In phage-antibiotic synergy (PAS), the application of a phage or phage cocktail with sublethal concentrations of antibiotics allows the virus to replicate in bacteria and prevent the further growth of microorganisms (10).

The Shiga toxin producing *E. coli* O157 strain is a global public health problem that causes a wide range of disorders from self-limited watery diarrhea and hemorrhagic colitis to life-threatening conditions as the hemolytic-uremic syndrome. Several large outbreaks of *E. coli* O157 were reported from the USA, Europe, Japan and Australia (13); *E. coli* O157 epidemics have also described in developing countries (14). Due to the resistance of pathogens and risk of developing hemolytic-uremic syndrome through induction of the expression of Shiga toxin, the chemotherapy of *E. coli* O157 infections is contraindicated and the infected person receives supportive treatments. Some therapeutic approaches are trying to treat *E. coli* O157 infections by Shiga toxin-binding resins (15), neutralization of Shiga toxins using a genetically-modified bacterium expressing the receptor of the toxin (16), a vaccine against the adhesion molecule, intimin, that is essential for persistent colonization in some animals (17), and phage therapy (2, 3, 18). 

This study aimed to evaluate the antibacterial action of a biocidal phage and beta-lactam antibiotics, particularly ampicillin, alone and in combination with a naturally-isolated phage. The synergistic action between a naturally isolated phage and ampicillin in the solid and liquid culture media was confirmed.

## Materials and Methods

***Bacterial strains***

*Escherichia coli *O157, used in this study, is a clinically isolated strain. This strain was verified using CHROMagarTM O157 differential medium (CHROMagar).*Escherichia coli* (ATCC 25922), *Pseudomonas aeruginosa* (ATCC 27853), *Proteus vulgaris *(ATCC 6380), *Proteus mirabilis*, and *Staphylococcus aureus* (ATCC 25923) was purchased from PADTAN TEB Co. (Tehran, Iran) and used for determination of the host range of the isolated phage.

***Phage isolation, propagation, and quantification***

Sewage and water samples from different places (West Azarbayjan province) were collected to find potential bacteriophages. For phage isolation, collected samples have been centrifuged at 15000 g (10 min at 4 ^°^C) then supernatants were filtrated by the 0.45 µm membrane filter and incubated with *E. coli* O157 for multiplying the required phages. To verify the existence of phages, 10 µl of the enriched samples were spotted onto a fresh layer of *E. coli *O157 cultured on the top agar plate. Each clear plaque was suspended in sterile SM buffer (NaCl 11 Mm and MgSO_4_•7H_2_O 8 mM in the Tris-HCl 50 mM, pH 7.5) to isolate lytic phage particles. Phage suspensions were diluted and mixed with *E. coli *O157 in the overlay agar method to obtain single phage plaques for subsequent phage extraction. The cycle of phage dilution and isolation was repeated until the consistent plaques were formed by each phage.

Plaque forming unit (PFU) of the isolated phage was measured using a modified overlay plate agar method by adding of 100 μl of 0.5% TTC (2,3,5-triphenyl tetrazolium chloride, Merck, Germany) to one ml of molten 0.5% agar (11). This modification resulted in clear plaques over the reddish background of the bacterial lawn after overnight incubation. The isolated phage was then used with a definite MOI (multiplicity of infection) in the experiments.

***Morphological and molecular characterization of the isolated phage***

A drop of isolated phage suspension was mounted on the Formvar-coated copper grid, the excess of the sample removed with filter paper and the prepared thin film subjected to negative staining as per the standard procedures. Grids were observed under a transmission electron microscope (TEM, Philips Bio Twin, CM100, Netherlands), at an accelerating voltage of 75KV. 

Phage genome was extracted by the standard method of the phenol-chloroform extraction. The purified nucleic acid was treated with DNase I and RNase A (Thermo Scientific Co ). For restriction analysis; the enzymes were added to the extracted genome and treated nucleic acids evaluated using 1% agarose gel. The proteins of the phage capsid were examined using SDS-PAGE according to the Laemmli method (19, 20). Purified phages were precipitated by ice-cold acetone. Precipitated proteins were collected by centrifugation (16,000×g, 20 min, 4 ^°^C), and resuspended in the sample buffer. The samples were then electrophoresed on 10% SDS-PAGE slab gel.

***Determination of the bacterial host range of the isolated phage***

The double-layer agar test was used to determine the ability of the phage to infect other bacterial strains. Bacterial cells were added to 3 mL of liquefied agar and poured on solid agar to prepare double-layer agar plates. A volume of 10 µl of the phage was applied on each bacterial lawn. After overnight incubation at 37 ^°^C, the formation of spots (zone of inhibition) was recorded as a positive result indicating the susceptibility of examined bacteria to the lytic phage. Control (negative) samples were done with buffer instead of the phage.

***Determination of antibiotic and phage susceptibility of E. coli O157***

The susceptibility of the *E. coli *O157 to beta-lactams including penicillin (P, 10 μg), amoxicillin (AMX, 25 μg), ampicillin (AM, 10 μg), cloxacillin (CX, 1 μg), amoxicillinclavulanate (AMC, 30 μg) was determined by disk diffusion method according to the M2-A8 protocol-CLSI 2014 using *Escherichia coli* (ATCC 25922) as quality control (21) and modified overlay assay method. Discs of the mentioned contents were obtained from PADTAN TEB Co. (Tehran, Iran). Briefly, the bacterial sample was standardized to equal 5×107 CFU/ml and overlaid on the Muller Hinton agar plates. In the overlay method, 100 μl of the bacterial suspension was mixed with the 3 ml of molten Muller Hinton agar and applied on prepared agar plates. Antibiotic disks were applied and plates incubated at 37 ^°^C overnight.

The MIC of ampicillin (Daana Pharma Co., Iran) for *E. coli* O157 were estimated by the broth macrodilution and microdilution method according to the M07-A9 protocol-CLSI 2014 (21). The inoculated tubes were incubated at 37 ^°^C for 16 to 18 hr. The MIC of ampicillin was defined as the lowest concentration that inhibited the visible growth of the organism. 

To evaluate the synergistic inhibitory effect of the phage and ampicillin disk, 10 µl of the phage preparation (PFU 10^9^) was mixed with 100 µl of the bacterial sample at MOI 2 (22). The rest of the steps including plating and applying the disks was done as aforementioned. Blank disks used as the negative controls (antibiotic and phage free control). Each experiment was repeated in triplicate fashion to ensure reproducibility. To improve the viewing and to interpret the results, both tests were also performed by adding 100 µl of 0.5% TTC solution to 1 ml of melted Muller Hinton agar in the method above. 

***Phage-antibiotic synergism using microtiter plate assay***

Antibacterial synergistic activity was also assigned by modified broth microdilution method in flat-bottomed 96 multi-well microplates. A total of four types of experimental conditions including ampicillin-alone, phage-alone, ampicillin-phage mix, and bacterial control at the final volume of 300 µl was designed. Briefly, the culture was allowed to grow until an OD_600_ of 0.15 reached 5×10^7^ CFU. Then 100 µl of the bacterial suspension was added to each well (final concentration of 10^7^ CFU in each well), and 100 µl of ampicillin (final concentrations of 1.562, 3.125, 6.25, 12.5 and 25 µ/ml) pipetted into ampicillin-alone and ampicillin-phage mix wells. To evaluate the values in the presence of phage gT0*E.co*-MGY2, the phage-alone and ampicillin-phage mix wells were infected at MOI of 10 by the administration of 100 µl of phage preparation (10^9^ PFU). Following to overnight incubation at 37 ^°^C, 50 μl of 0.1% filter-sterilized TTC (Sigma Chemical Co., St. Louis, MO, USA) was added into each well and incubated for additional 2 hr. The absorbance was read at 540 nm using a plate reader (Biotech, Vantaa, Finland). 

***Kinetics of antibacterial activity of ampicillin and phage***

To assess the effects of the isolated phage on the bacterial growth curve in the large-scale liquid medium, *E. coli *O157 was cultured at 37 ^°^C in 100 ml of LB broth (Lauria Bertani, Merck, Germany) in a shaking incubator (120 rpm). When the culture reached OD600 of 0.15 (~5×107 CFU), the bacteria were aliquoted into identical flasks (final concentration of 107 CFU). Then, 1 ml of ampicillin (final concentrations of 1.562, 3.125, 6.25, 12.5 and 25 μ/ml) and the phage at MOI of 10 (final PFU of phage in flasks was 108) were added to the corresponding flask. Samples (1 ml) of the bacterial culture were hourly taken, and the OD of tests and control cultures were measured at 600 nm for 6 hr. All experiments were done in triplicate fashion.

***Statistical analysis***

All experiments were performed in replicates. Data were analyzed using the Minitab software. The one-way ANOVA and Fisher’s least significant difference (LSD) posttest were used to compare means of treatments, including the control, ampicillin, phage, and mix (ampicillin and phage). Mean differences among treatments were considered significant if *P*<0.05.

## Results

***Phage isolation, morphology and host range***

Screening of the sewage from different wastewater plants of West Azerbaijan province in Iran yielded phages with various levels of lytic activity against *E. coli* O157. The bacteriophage content of samples was initially amplified by an enrichment phase. From which one specific bacteriophage against *E. coli* O157, designated as gT0*E.co*-MGY2, was purified from a single clone (clear zone) through successive plaque-picking steps using plaque formation technique on double-layer agar plates. Final selection of the most promising candidate for the lytic ability was made based on the relative size and clarity of plaques. The environmental sewage collected from an urban slaughterhouse was the original source of the studied phage. The phage was propagated in the host cell strain and stored at 4 ^°^C for further analysis. 

**Figure 1:**
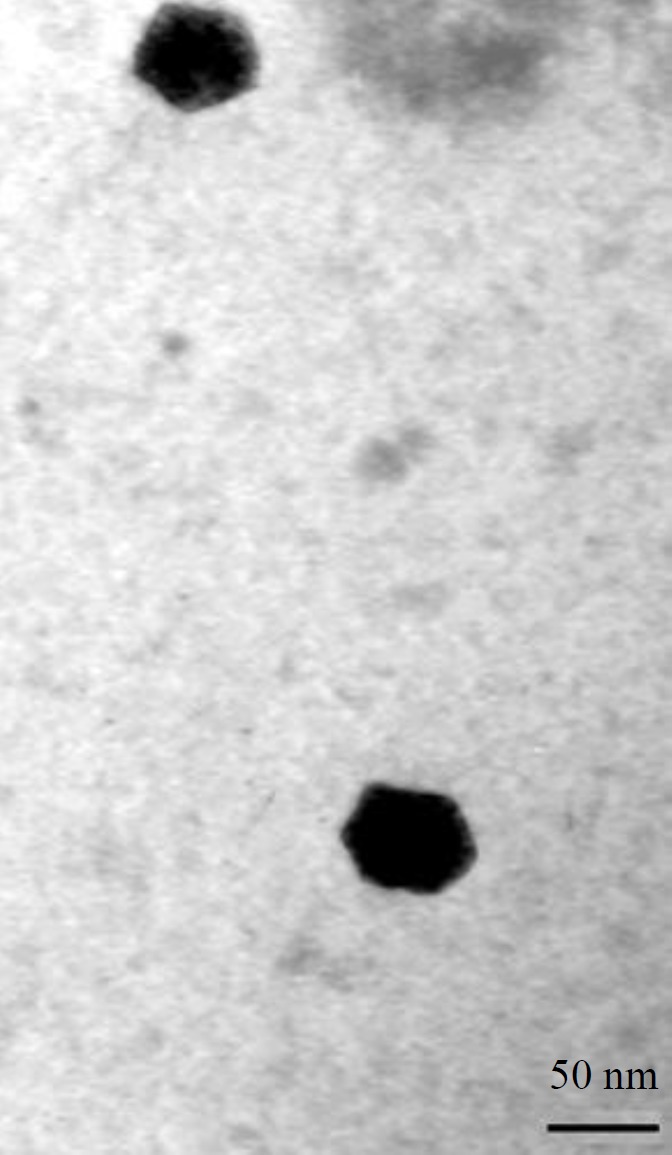
Electron micrograph of phage gT0E.co-MGY2 particles under a transmission electron microscope. Scale bar represents 50 nm

**Figure 2:**
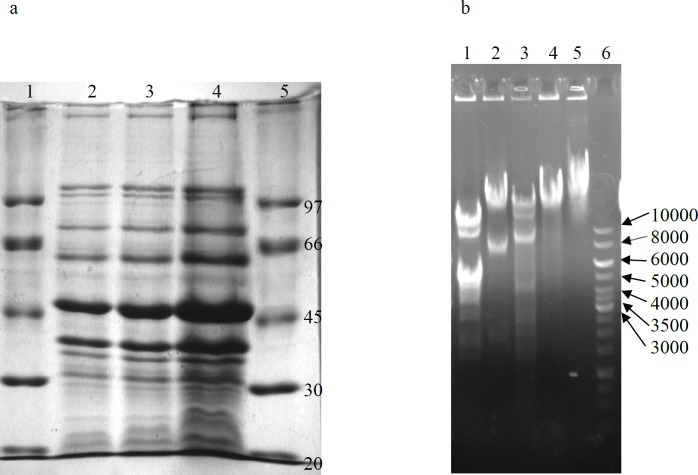
Proteome of the phage gT0E.co-MGY2 (a). Lane 1, size marker, lane 2, 3 and 4 acetone precipitated phage protein with different concentration. Restriction analysis of the genome of the phage gT0E.co-MGY2 (b). Lane 1, 2, 3, and 4 treated genomes with *Sau*3A, *Hin*DIII, *Eco*RI, *Bam*H1, respectively, lane 5, extracted genome, lane 6, DNA ladder

Based on transmission electron microscopy (TEM), the isolated phage is a tail-less icosahedral particle with ~50 nm diameter (Figure 1). Genome purification of the phage and subsequent treatment with RNase, DNase, and restriction analyses revealed that it contains a dsDNA genome. SDS-PAGE (10%) analysis of the phage proteins resulted in a band pattern shown in Figure 2. The major band of the phage proteins is ~47 kDa. Other dominant protein bands on SDS-PAGE are seen at approximately 38 and 63 kDa, which may be attributed to spike and receptor binding proteins of the phage (20). 

The effectiveness of phage gT0*E.co*-MGY2 to kill other bacteria such as *Escherichia coli* (ATCC 25922), *Pseudomonas aeruginosa* (ATCC 27853), *Proteus vulgaris* (ATCC 6380), *Proteus mirabilis,* (ATCC 7002) and *Staphylococcus aureus *(ATCC 25923) was evaluated by plaque formation and spot test. Based on experiments, the host range of the phage was limited to *E. coli* O157, and the virion was not able to kill other bacterial strains.

***Antibacterial activity of the isolated phage***

**Figure 3:**
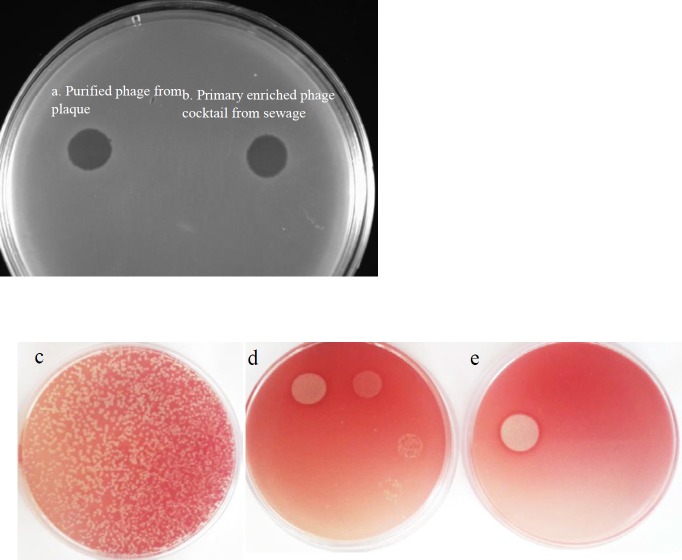
Inhibition zones on the *Escherichia coli *O157 lawn. (a) the left spot is pertaining to purified phage from single plaque and (b) the right spot was produced by the phage cocktail enriched from sewage. Determination of plaque forming unit by serial dilution of the phage and evaluation of clear zones produced by spot test on TTC mixed overlay agar plates, (c) plaque forming units of phage at MOI of 10, (d) spotting serial dilution of the phage (10 µl ) on TTC mixed bacterial lawn, (e) spot test of concentrated phage

The turbidity comparison of bacterial culture inoculated by the phage and phage-free control culture determined the lytic activity of the isolated phage. However, to more carefully assess the killing action of the phage against target bacteria, antimicrobial challenge tests including spot assays with or without TTC were performed and the clear zones of inhibition observed with no colonies grown in the area of halos (Figure 3). These spots on the bacterial lawn confirmed the lytic and biocidal activity of the phage and its potential antibacterial effect. 

***Synergistic action of the antibiotic and phage in modified disk diffusion method***

**Figure 4:**
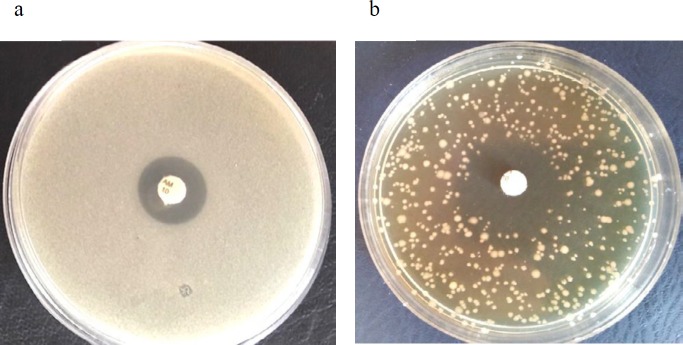
Plate (a) shows the antibiotic susceptibility of bacteria against ampicillin in disk diffusion test, plate (b) indicates the same experiment in the presence of 10 µl of the phage preparation (PFU 10^9^) mixed with bacteria at MOI 2

**Figure 5:**
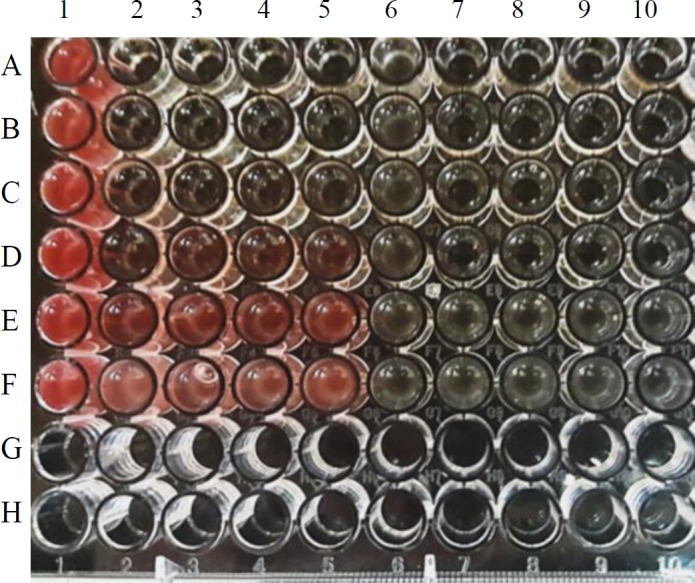
Synergistic action of the phage and ampicillin in the microdilution assay. The image of the plate in the microdilution method, TTC reduction by live bacterial cells changed the color to red, which is correspondent to the bacterial growth. Column 1, rows A-F: Bacterial control (CFU 107) test repeats. Columns 2-5: Ampicillin-alone tests, rows A-F: ampicillin at concentrations of 50, 25, 12.5, 6.25, 3.125, and 1.562 µg/mL, respectively. Column 6, rows A-F: Phage-alone test repeats are. Columns 7-10, rows A-F: Phage-ampicillin combinations (including phage at MOI 10 and ampicillin at the same concentrations of column 2-5). The rows G-H are empty

According to the inhibition zone diameters in the CLSI 2014 susceptibility assays (21), *E. coli* O157 used in the study was resistant to penicillin, cloxacillin, ampicillin, amoxicillin and had intermediate susceptibility to the amoxicillin-clavulanic acid combination. Applying phage gT0*E.co*-MGY2 to the bacterial lawn of disk diffusion assays changed the size of clear zones of ampicillin and amoxicillin-clavulanic acid considerably as well as the overall growth of the bacterial culture (Table 1 and Figure 4). Phage-containing plates (MOI 10) showed expanded inhibition zones for the above-mentioned antibiotics. These larger zones of inhibition were accompanied by limiting bacterial growth on the phage-containing plates and disperse bacterial colonies were observed instead of dense bacterial lawn in the phage-free agar overlay plates (Figure 5). Adding the phage to disk diffusion experiments, according to observed diameters of inhibition zones and CLSI susceptibility criteria, changed the status of susceptibility from resistant to sensitive in the case of ampicillin and from intermediate to sensitive in the case of amoxicillin-clavulanic acid (21). This is the first report on the synergistic action between a naturally isolated phage and ampicillin. Moreover, the modified disk diffusion assay was developed in this study can be used to detect the synergy between phage and antibiotics. Additive effects were not detected with other tested beta-lactams including penicillin and cloxacillin. 

***Phage and antibiotic synergism in the endpoint microtitration assay***

**Figure 6:**
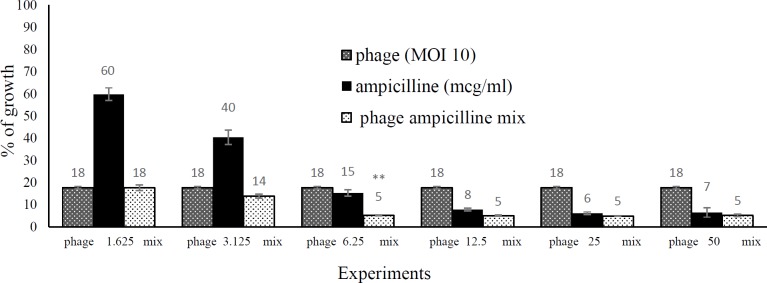
Synergy between the phage and ampicillin in the endpoint microdilution assay. The percentage of the growth of *Escherichia coli *O157 in the presence of ampicillin at concentrations of 1.562, 3.125, 6.25, 12.5, 25 µg/ml and by the same concentrations mixed with the phage at MOI 10; the incubation time was 6 hours at 37 ^°^C

The MIC value for ampicillin was 50 µg/mL against *E. coli* O157 in the microdilution and macrodilution method. The synergistic activity of phage gT0*E.co*-MGY2 and ampicillin was further investigated by measuring the bacterial growth in 96-well microplates following the addition of TTC. The absorbance was recorded and compared between ampicillin-alone (50, 25, 12.5, 6.25, 3.125 and 1.562 µg/ml), phage-alone (MOI of 10) and phage-ampicillin combinations (Figures 5 and 6). In low concentrations, ampicillin was not effectively inhibited the growth of bacteria (40% for 1.562 and 60% for 3.125 µg/ml), and in high ampicillin concentrations, there was no significant difference in inhibition percentage of bacterial growth between ampicillin-alone and phage-ampicillin combination experiments (92%, 94% and 93% for ampicillin 50, 25 and 12.5 µg/ml of and 95% for all phage-ampicillin combinations). However, the ampicillin-phage mixture (95% growth inhibition) was significantly more potent than phage-alone (85% growth inhibition) and antibiotic-alone (82% growth inhibition) at 6.25 µg/ml of ampicillin. According to the growth inhibition percentages calculated after 6 hr incubation, the synergistic interaction of phage gT0*E.co*-MGY2 and ampicillin at a concentration of 6.25 µg/ml (of MIC) was the highest (Figures 5 and 6), where the phage-ampicillin combination produced antibiotic effect significantly (*P*-value 0.05) greater than any of them individually.

***Kinetics of synergistic antibacterial activity of the phage and ampicillin***

**Figure 7:**
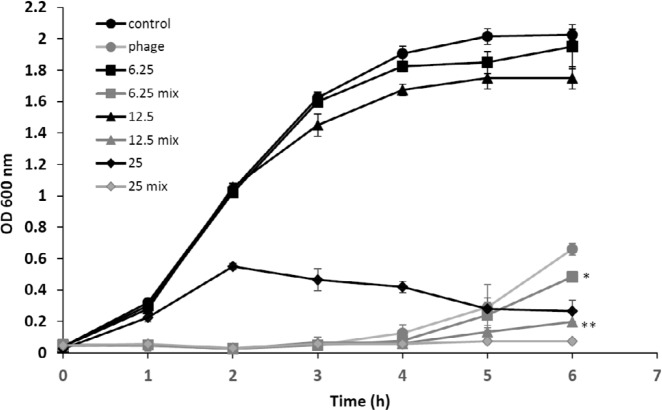
Effect of the synergistic antibacterial activity of the phage and ampicillin on the *Escherichia coli *O157 growth curve

The antimicrobial effect of ampicillin-alone (25, 12.5, 6.25 µg/ml), phage-alone (MOI of 10), and combination (ampicillin and phage) was evaluated for 6 hr to obtain growth kinetics curve as displayed in Figure 7. Compared to the control, ampicillin at a dose of 25 µg/ml, and the phage (MOI 10) significantly inhibited the growth of *E. coli* O157, while the concentration of 6.25 µg/ml (of MIC) and 12.5 µg/ml ( of MIC) of ampicillin led to a slight change in the growth curve. The action of subinhibitory concentrations of ampicillin initiated in few hours after inoculation. However, the phage gT0*E.co*-MGY2 exhibited immediately significant lytic activity, which lasted for 6 hr of treatment in *in vitro* culture conditions (MOI 10, CFU 10^7^). At the first 4 hr, the rate of bacterial growth in phage-alone experiments was strictly limited, and the curve remained nearly constant at initial OD value, but then increased slightly at the sixth hour of the experiment that can be explained by emerging resistant bacteria.

To avoid the growth of phage-resistant strains, we attempted to co-administer the isolated phage with ampicillin. Combination of the phage and sublethal concentrations, 6.25 µg/ml (of MIC) and 12.5 µg/ml (of MIC), of ampicillin more effectively (*P*-value of 0.05 and 0.001, respectively) inhibited the growth of *E. coli* O157 than antibiotic- or phage-alone and the recovery rate of resistant strain was considerably reduced in combination (Figure 7). It can be postulated that phage-antibiotic synergy might be impeded the development of resistant strains. 

**Table 1:** Inhibition zone for different antibiotic disks in the presence and absence of the phage gT0*E.co*-MGY2. Penicillin (P), cloxacillin (CX), ampicillin (AM), amoxicillin (AMX), amoxicillin-clavulanic acid (AMC)

Antibiotic (µg)	The diameter of inhibition zone (mm)			Change in susceptibility level
	Without phage		With phage	
P (10)	6±0.5	9±0.5		-
CX (1)	6±0.5	6±0.5		-
AM(10)	12.6±1.2	27±2.5		Resistant to sensitive
AMX(25)	9±1	12±1		-
AMC (30)	16±0.5	23±2		Intermediate to sensitive

## Discussion

Recently, phage therapy has been reemerging as a highly promising alternative management against antibiotic-resistant bacteria. Phage therapy has some advantages over the antibiotic treatment. Phages are unique magic bullets, and most of them infect and kill a narrow spectrum of bacteria or a particular strain (23). 

In a screening set-up, spot testing and plaque assay were used to identify suitable bacterial virus for potential application in phage therapy as well as the determination of host range. Mixing TTC to agar plates in spot test and plaque assay made it easy to view the spots and count plaques with the naked eye for measuring plaque forming units (PFU) of the phage (Figure 4). Phage gT0E.co-MGY2 is specific for *E. coli* O157 and do not infect other *E. coli* strains and none of the other pathogenic bacteria. This narrow host-range feature found in most bacteriophages is a benefit for probable clinical application of the phage which could avoid dysbiosis and decrease the risk of developing secondary infections which occur due to the removal of normal flora (25). According to its morphological and molecular characterizations, and classification system of Ackermann, this phage was assigned to the family tectiviridae (25), which are natural infecting bacteriophage for Gram-negative and -positive bacteria. Relatives of phage gT0E.co-MGY2 in the tectiviridae family have no head-tail structure, but the ability to produce tail-like spikes serving as a DNA injection tool. Phage gT0E.co-MGY2is an icosahedral phage with a diameter of about 90 nm, which is similar to other tectiviral phages. 

In a modified disk diffusion method, we investigated whether the presence/absence of phage could affect inhibition zones of penicillins. Ampicillin and amoxicillin-clavulanic acid antibacterial action potentiated by the phage and the size of the clear zones of the ampicillin disk increased by more than double that of normal disk diffusion value at MOI of 10. In a similar study, applying the phage against *S. aureus* yielded a 10% increase in the case of ciprofloxacin at MOI of 1 (25). The sublethal concentration of ampicillin particularly dose of 6.25 µg/ml was the optimal concentration for effective phage-antibiotic synergy. This synergistic effect has been previously reported for *E. coli* (12), *S. aureus* (26), *P. aeruginosa* (27), *Burkholderia cepacia* (28), and *Proteus mirabilis* (29) with antibiotics including ciprofloxacin, cefotaxime, meropenem, and tetracycline. But, has not been described for penicillins antibiotics so far. 

The MIC values for ampicillin was determined 50 µg/mL and the highest decrease in the bacterial growth was observed at 1/8 of this value (6.25 µg) using combined antimicrobial treatment in the microtiter plate format. The modified broth microdilution assay confirmed the PAS of ampicillin and the phage, which initially detected by altered disk diffusion test, proposing this method can be used as primary screening for recognition of PAS. Based on the growth curve, the bacteriophage component stopped the bacterial proliferation from the beginning in the cases of phage-only and phage-ampicillin combination, and this can likely compensate the delayed onset of antibiotic action in the standard anti-infective therapies and can help the host immune system eliminate the pathogen in the early phase of infection. The results of growth curve experiments also confirmed the synergistic effect of the phage and ampicillin at sub-MIC concentrations and can be suggested as an effective way to attenuate the incidence of phage- and antibiotic-resistant *E. coli* O157. 

Several mechanisms may be involved in the development of phage-antibiotic synergistic effect. This potentiation may result from stimulation of lytic phage growth in the presence of beta-lactams (9). Cell filamentation is a morphological change that is induced in the presence of subinhibitory concentrations of beta-lactams such as ampicillin. Inhibiting synthesis of the cell wall and cell division by beta-lactams is the cause of cell filamentation. Morphological alteration of the cells assists assembly of phages by escalating the essential precursors for maturing of bacteriophages and easing the cell for lysis (9). Therefore, it could result in an increased burst size and reduced latent period of the bacteriophages. Another proposed mechanism for this synergistic effect is structural modifications produced by the addition of bacteriophage, restricting the capability of the bacteria to resist antibiotic exposure. Lytic bacteriophages specifically bind to one or more surface receptors on the bacterial hosts (30). Some of these surface receptors are efflux pumps that drive antibiotics from the bacterial cytoplasm into external media and cause resistance to chemical agents. Occupation of these receptors changes the efflux pump function, leading to increased susceptibility to antibiotics (31). 

## Conclusion

Phages can evolve or even co-evolve along with the bacteria, and consequently restrict the evolution of resistance mechanisms in the pathogen during the course of management. However, developing the phage-resistant strains (as well as antibiotic-resistance strains) is inevitable because bacteria eventually adapt and generate different survival strategies. The rationality behind the co-administration of bacteriophages and antibiotics arise from an evolutionary conception that two sufficiently different selective pressures are likely to be more effective than either alone , and likely lowers the chance of the occurrence of resistance mutations as shown in this study. The co-administration of subinhibitory concentrations of ampicillin in mixture with the natural phages can also result in avoidance of antibiotic side effects arising from employing high doses. Finally, it should be considered that for safely use of phages as alternatives or supplement to antibiotics establishments of the quality and safety standards for human treatment is needed. 
